# Local and systemic glucocorticoid metabolism in inflammatory arthritis

**DOI:** 10.1136/ard.2008.090662

**Published:** 2008-04-17

**Authors:** R Hardy, E H Rabbitt, A Filer, P Emery, M Hewison, P M Stewart, N J Gittoes, C D Buckley, K Raza, M S Cooper

**Affiliations:** 1 Division of Medical Sciences, University of Birmingham, Queen Elizabeth Hospital, Edgbaston, Birmingham, UK; 2 Rheumatology, University of Birmingham, Queen Elizabeth Hospital, Edgbaston, Birmingham, UK; 3 Academic Section of Musculoskeletal Disease, Leeds Institute of Molecular Medicine, University of Leeds, Leeds, UK; 4 Department of Orthopedic Surgery, UCLA-Orthopedic Hospital, Los Angeles, California, USA

## Abstract

**Background:**

Isolated, primary synovial fibroblasts generate active glucocorticoids through expression of 11β-hydroxysteroid dehydrogenase type 1 (11β-HSD1). This enzyme produces cortisol from inactive cortisone (and prednisolone from prednisone).

**Objective:**

To determine how intact synovial tissue metabolises glucocorticoids and to identify the local and systemic consequences of this activity by examination of glucocorticoid metabolism in patients with rheumatoid arthritis (RA).

**Methods:**

Synovial tissue was taken from patients with RA during joint replacement surgery. Glucocorticoid metabolism in explants was assessed by thin-layer chromatography and specific enzyme inhibitors. RT-PCR and immunohistochemistry were used to determine expression and distribution of 11β-HSD enzymes. Systemic glucocorticoid metabolism was examined in patients with RA using gas chromatography/mass spectrometry.

**Results:**

Synovial tissue synthesised cortisol from cortisone, confirming functional 11β-HSD1 expression. In patients with RA, enzyme activity correlated with donor erythrocyte sedimentation rate (ESR). Synovial tissues could also convert cortisol back to cortisone. Inhibitor studies and immunohistochemistry suggested this was owing to 11β-HSD2 expression in synovial macrophages, whereas 11β-HSD1 expression occurred primarily in fibroblasts. Synovial fluids exhibited lower cortisone levels than matched serum samples, indicating net local steroid activation. Urinary analyses indicated high 11β-HSD1 activity in untreated patients with RA compared with controls and a significant correlation between total body 11β-HSD1 activity and ESR.

**Conclusions:**

Synovial tissue metabolises glucocorticoids, the predominant effect being glucocorticoid activation, and this increases with inflammation. Endogenous glucocorticoid production in the joint is likely to have an impact on local inflammation and bone integrity.

Since the discovery of cortisone and its first use in patients with rheumatoid arthritis (RA)^1^ glucocorticoids have been extensively used to suppress synovial inflammation. However, in patients with established synovitis, glucocorticoids such as cortisol (hydrocortisone), prednisone and prednisolone do not cause permanent resolution of inflammation and long-term use has adverse effects on bone, skin and fat tissue.^2^ ^3^ Endogenous glucocorticoids also have a role in suppressing disease activity in RA. Early morning stiffness is attributed to the nocturnal decrease in circulating cortisol levels. Administration of metyrapone to reduce endogenous corticosteroid production increases disease activity in RA.^4^ It is unclear, however, whether endogenous corticosteroid action contributes to susceptibility to, or severity of, RA. Subtle abnormalities of the hypothalamic-pituitary-adrenal axis have been seen in glucocorticoid-naive patients with RA^5^^–^7 but their origin remains unclear.^8^

We have previously hypothesised that periarticular osteopenia in RA is partly due to excessive local glucocorticoid activation through the 11β-hydroxysteroid dehydrogenase type 1 (11β-HSD1) enzyme.^9^ This enzyme converts inactive steroids (cortisone and prednisone) to their active counterparts (cortisol and prednisolone).^10^ Although 11β-HSD1 is bidirectional, its predominant action in vivo is conversion of inactive to active glucocorticoids. Hepatic 11β-HSD1 is essential for activation of oral cortisone/prednisone—patients who lack this enzyme are unresponsive to cortisone and prednisone but respond to hydrocortisone and prednisolone.^11^ We have reported that synovial fibroblasts express 11β-HSD1 in vitro and in vivo.^12^ In osteoblasts and synovial cells 11β-HSD1 activity is upregulated by proinflammatory cytokines.^9^ ^12^ This suggested that 11β-HSD1 might generate high levels of glucocorticoids within the joint and that this might contribute to periarticular osteopenia.

By contrast, a related enzyme 11β-HSD2 solely inactivates steroids. This enzyme is expressed in mineralocorticoid target tissues, various developmental tissues and some tumours.^13^^–^15 Recent studies have reported expression of 11β-HSD2 in peripheral blood mononuclear cells (PBMCs) and synovium of patients with RA.^16^^–^18 We therefore examined glucocorticoid metabolism and function in synovial tissue from patients with RA using specific enzyme assays and inhibitors. In addition, we examined glucocorticoid concentrations in synovial fluid and compared the systemic metabolism of glucocorticoids in patients with RA and non-inflammatory joint conditions.

## PATIENTS AND METHODS

### Patients

Biopsy specimens of matched synovium and skin were obtained during hip, knee or elbow arthroplasty from consenting patients who fulfilled the American College of Rheumatology criteria for RA and OA. Table 1 gives clinical details of the patients.

**Table 1:** Clinical characteristics of subjects for synovial tissue corticosteroid metabolism studies

Patients	Age (years)Mean (SD)	F/M (n)	Site of operation (n)	Treatment (n)	ESR(mm/1st h)Mean (SD)	CRP(mg/l)Mean (SD)
With RA (n = 12)	62 (10)	11/1	Hip (6) Knee (4) Elbow (2)	Methotrexate (3) Prednisolone (3) Anti-TNF (2) Sulfasalazine (1) Hydroxychloroquine (1) Azathioprine (1)	39 (20)	27 (23)
						
With OA (n = 8)	67 (7)	6/2	Hip (7) Knee (1)		14 (11)*	

*p<0.05 compared with patients with rheumatoid arthritis.

CRP, C-reactive protein; ESR, erythrocyte sedimentation rate; OA, osteoarthritis; RA, rheumatoid arthritis; TNF, tumour necrosis factor.

Synovial tissue was taken on ice and prepared within 2 h by removing adherent non-synovial tissue. Tissue was divided into 100 mg sections for enzyme assay or ELISA. Skin tissue was prepared by removing subcutaneous fat and dividing into 100 mg pieces.

Matched synovial fluid and serum samples were obtained from patients with active RA undergoing joint aspiration as part of routine care. Blood was drawn immediately before joint aspiration. Clinical details are given online in supplementary table 1.

Urine samples for corticosteroid metabolite analysis were obtained from patients with newly presenting RA or non-inflammatory joint disease (localised OA (n = 5); trigger finger (n = 3); hypermobility (n = 1)). Clinical details are given in supplementary table 2.

All studies had ethical approval from the local ethics committee and informed consent was obtained when samples were taken.

### 11β-Hydroxysteroid dehydrogenase enzyme assays

Synovial or skin tissue (100 mg per assay) was incubated in RPMI-1640 medium containing 1% non-essential amino acids, 1% penicillin/streptomycin, 1% sodium pyruvate, 2 mM glutamine and 20% heat-inactivated fetal calf serum (FCS; Labtech International, Sussex, UK). Cortisol (100 nM; to measure glucocorticoid inactivation) or cortisone (to measure activation) along with tracer amounts of [3H]cortisol (specific activity 78.4 Ci/mmol; NEN Life Science Products, Hounslow, UK) or [3H]cortisone (generated as previously described^19^) were added and tissue incubated at 37°C for 18 h. Similar assays were performed using [3H]prednisolone or [3H]prednisone.^20^ Steroids were extracted from medium/tissue using dichloromethane (5–7 ml) and separated by thin-layer chromatography using ethanol:chloroform as the mobile phase. Thin-layer chromatography plates were analysed using a Bioscan imager (Bioscan, Washington DC, USA) and fractional steroid conversion calculated. Results were expressed as pmol product/mg tissue/h. Experiments were carried out in duplicate or triplicate.

### RNA extraction and reverse transcription

RNA was extracted from synovium using a single-step method (TRI Reagent, Sigma, Poole, UK). Aliquots (1 μg) of RNA were reverse transcribed using random hexamers in a 20 μl volume according to the manufacturer’s protocol (Promega, Madison, USA).

### Real-time PCR

Expression of mRNA for 11β-HSD1/2 was assessed by real-time PCR in an ABI 7500 system (Applied Biosytems, Warrington, UK). Reactions were performed in 25 μl aliquots on a 96-well plate (Sigma). Primers for 18S were used as an internal reference. Reactions contained TaqMan PCR master mix (Applied Biosytems), 900 nmol primers, 100–200 nmol TaqMan probe and 25–50 ng cDNA. Reactions were as follows: 50°C for 2 min, 95°C for 10 min, 44 cycles of 95°C for 15 s and 60°C for 1 min. Data were obtained as Ct values (cycle number at which logarithmic PCR plots cross a calculated threshold line) according to the manufacture’s guidelines, and used to determine ΔCt values (Ct of target gene – Ct of housekeeping gene) as raw data for gene expression. Probe and primer sequences were 11β-HSD1:

forward AGGAAAGCTCATGGGAGGACTAG,  reverse ATGGTGAATATCATCATGAAAAAGATTC,  probe CATGCTCATTCTCAACCACATCACCAACA;  11β-HSD2: forward CAGGTGTCCTAGTGCACATTGAC,  reverse GTAGCCCACTCTCTCGTCCAA,  probe AAGGCACGCCCTCCCAGCG.

### Immunohistochemistry

Cryostat sections of synovial tissue were analysed with previously validated sheep polyclonal antibodies to 11β-HSD1 and 11β-HSD2 1/50 (The Binding Site, Birmingham, UK)^14^ ^21^ with anti-sheep/goat biotin AB360 as secondary antibody 1/50. Immunohistochemistry was also carried out using polyclonal antisera to the endothelial marker von Willebrand’s factor 1/1000 (Dako, Ely, UK); the fibroblast marker ASO2 (CD90) 1/100 (Invitrogen, Paisley, UK); and the monocyte/macrophage marker CD68 1/100 (BD Biosciences, New Jersey, USA). Immunohistochemical analyses were carried out as described previously.^12^

### Measurement of corticosteroid levels in serum and synovial fluids

Serum and synovial fluid cortisol and cortisone levels were measured using a specific cortisol ELISA (R&D Systems, Abingdon, UK) and a previously reported radioimmunoassay for cortisone.^22^ Since serum and synovial fluid samples contain cortisol-binding proteins these techniques only give information about total cortisol concentration (rather than unbound “free” levels).^23^ Because there is much less protein binding of cortisone, levels of cortisone ascertained by these methods are more likely to reflect “free” levels.^22^

### Measurement of urinary corticosteroid metabolites

The gas chromatography–mass spectrometry method was based on Palermo *et al*^24^ using a 5970 mass spectrometer (Hewlett-Packard, Houston, Texas, USA). Intra-assay and interassay coefficients of variation were <10% for cortisol and cortisone. 11β-HSD1 activity was calculated as the tetrahydrocortisol + allo-tetrahydrocortisol/tetrahydrocortisone ((THF+alloTHF)/THE) ratio. Renal 11β-HSD2 activity was calculated as the urinary-free cortisol/cortisone ratio (UFF/UFE). The sum of total corticosteroid metabolites (THF, alloTHF, THE, cortols, cortolones, UFF, UFE) was used as an index of total cortisol secretion.

### Analysis of interleukin 6 (IL6) levels by ELISA

Soluble IL6 in tissue supernatants was measured using a sandwich ELISA (BD Biosciences Pharmingen, Torreyana, San Diego, USA). The limit of detection was 2.2 pg/ml and intra-assay and interassay coefficients of variation were 5.2% and 9.3%. Data were expressed as pg IL6/mg tissue.

### Statistics

Data were reported as the mean (SD) of replicate mean values for separate synovial explants unless otherwise stated. Regression analysis was performed using Microsoft Excel 2003. One-way analysis of variance was performed using SPSS Data Editor.

## RESULTS

### Enzyme activity studies

**Figure 1:**
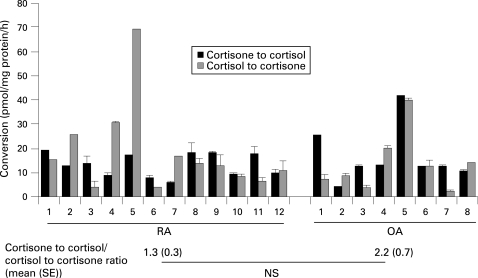
Glucocorticoid metabolism in synovial explants taken from patients with rheumatoid arthritis (RA) and osteoarthritis (OA). The ability of synovial tissue to interconvert cortisone and cortisol was examined using tissue freshly isolated after joint replacement surgery. Data are steroid generation for each sample adjusted for protein content and assay time and are expressed as mean (SD) of samples from each patient.

**Figure 2:**
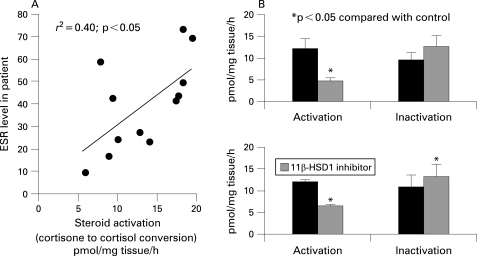
Relationship between glucocorticoid activating capacity of synovial explants and the systemic inflammatory response and the effect of a specific inhibitor of 11β-hydroxysteroid dehydrogenase type 1 (11β-HSD1) on glucocorticoid activation and inactivation. (A) A significant correlation was seen between glucocorticoid activation (oxoreductase activity) and the erythrocyte sedimentation rate (ESR) measured before surgery in patients with rheumatoid arthritis (RA). (B) The effect of PF-877423, a specific bidirectional inhibitor of 11β-HSD1 enzyme activity, on glucocorticoid activation (oxoreductase activity, cortisone to cortisol conversion) and inactivation (dehydrogenase activity, cortisol to cortisone conversion) in synovium from patients with RA or osteoarthritis (OA). The inhibitor reduced the capacity of synovium to activate glucocorticoids but was unable to block the inactivating capacity. In OA tissue inactivation increased with 11β-HSD1 inhibition. These results indicate that the inactivating capacity is not due to 11β-HSD1.

11β-HSD enzyme activity was present in all synovial samples from patients with RA (fig 1). Activity was also present in synovium from patients with osteoarthritis (OA). Activity was bidirectional in all cases, although the relative amounts of activation (cortisone to cortisol conversion) to inactivation (cortisol to cortisone) varied between subjects. There was no significant difference between RA and OA synovium in overall levels of each activity or in the ratio of activation to inactivation. Compared with patient-matched skin samples synovial samples had greater mean (SE) activating capacity (14.1 (1.8) vs 8.9 (1.3) pmol/mg protein/h; p<0.001) and a higher ratio of activation to inactivation (ratio 1.7 (0.4) vs 0.6 (0.1); p<0.01), but there was no difference in inactivating capacity (16.1 (3.5) vs 20.1 (3.4); NS). For glucocorticoid activation in synovium there was a significant positive correlation with the preoperative erythrocyte sedimentation rate (ESR; fig 2A), but this relationship was not seen with CRP (*r*^2^ = 0.04, NS). Inactivation did not correlate with either inflammatory marker. Synovial tissue also metabolised prednisone and prednisolone in a manner indistinguishable from cortisone/cortisol (for 100 nM substrate activation assay: cortisone to cortisol 16.3 (4.7) pmol/mg protein/h, prednisone to prednisolone 23.0 (5.1); for inactivation assay; cortisol to cortisone 15.2 (6.1), prednisolone to prednisone 12.5 (4.7); n = 4, NS).

### Characterisation of glucocorticoid inactivating capacity

The glucocorticoid activating (oxoreductase) capacity of synovial tissue appeared to be due to expression of 11β-HSD1 as this is the only enzyme known to convert cortisone to cortisol. Additionally, we have previously localised 11β-HSD1 in synovium by immunohistochemistry^12^ and RT-PCR on synovial tissue demonstrated abundant 11β-HSD1 mRNA expression (Ct value 32.0 (0.7) relative to 12.9 (1.9) for 18S).

**Figure 3:**
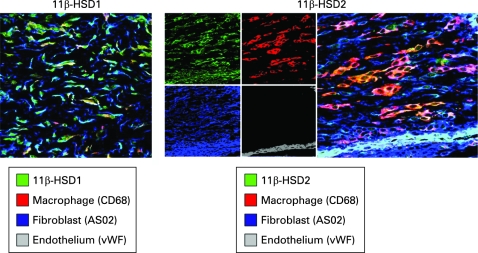
Immunohistological localisation of 11β-hydroxysteroid dehydrogenase (11β-HSD) enzymes in synovium. The expression of 11β-HSD1 and 11β-HSD2 within rheumatoid synovium were examined using specific antibodies. 11β-HSD1 expression colocalised predominantly with a fibroblast marker. Expression of 11β-HSD2 colocalised with expression of CD68.

The glucocorticoid inactivating (dehydrogenase) activity could be due to either 11β-HSD1, 11β-HSD2 or a combination of both. The lack of correlation between activation and inactivation (p = 0.56) suggested the presence of more than one enzyme and a specific inhibitor of 11β-HSD1 (PF-877423; Pfizer, New York, USA) was used to clarify this further. PF-877423 blocks both activating (oxoreductase) and inactivating (dehydrogenase) directions of 11β-HSD1 but has no effect on 11β-HSD2 activity (Bujalska *et al*, submitted for publication). Enzyme activity data indicated that the inhibitor reduced glucocorticoid activation in synovial tissue from patients with RA and OA but increased glucocorticoid inactivation in OA tissue (fig 2B). There was also a trend towards higher inactivation in RA tissue treated with inhibitor (p = 0.07). These observations suggested that glucocorticoid inactivation in synovial tissue was due to expression of 11β-HSD2 rather than 11β-HSD1. 11β-HSD2 was detectable by RT-PCR on RNA extracted from synovial tissue (Ct 33.6 (1.0) relative to 12.1 (1.8) for 18S). Immunohistochemistry for 11β-HSD2 demonstrated expression of the enzyme in both RA and OA tissue, where it colocalised predominantly with CD68-positive macrophages (fig 3). This contrasted with 11β-HSD1 expression which was localised primarily to fibroblasts.

### Functional consequences of enzyme activity

**Figure 4:**
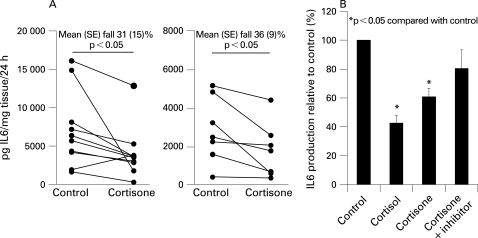
The functional effect of glucocorticoid activation on interleukin 6 (IL6) synthesis in patients with rheumatoid arthritis (RA) and osteoarthritis (OA). (A) Synovial tissue was incubated for 24 h in the presence or absence of 100 nM cortisone. In both RA and OA synovium cortisone had a significant suppressive effect on IL6 production. (B) A specific 11β-HSD1 inhibitor (PF-877423) was able to block the decrease in IL6 production caused by cortisone treatment.

High levels of IL6 were produced by synovial tissue. As expected, levels were higher in patients with RA than in those with OA. Incubation of synovial tissue with cortisol significantly reduced IL6 production, indicating tissue sensitivity to glucocorticoids. Incubation with cortisone also caused a significant reduction in IL6 expression, suggesting that synovial 11β-HSD1 was functionally active, converting cortisone to cortisol in an autocrine fashion (fig 4A). This was confirmed by studies using PF-877423, which blocked the suppressive effect of cortisone on IL6 (fig 4B).

### Measurement of tissue glucocorticoid levels in vivo

To evaluate net synovial tissue glucocorticoid metabolism within the joint we measured corticosteroid levels in paired serum and synovial fluid samples. To minimise the confounding effect of cortisol-binding proteins we measured serum-to-synovial fluid concentration gradients in cortisone since cortisone has limited binding to these proteins. Cortisone levels in synovial fluid obtained from patients with RA were significantly different from plasma levels (serum 56.7 (11.8) nmol/l, synovial fluid 20.9 (8.7) nmol/l; mean fall from serum to synovial fluid 63%, p<0.001). The low cortisone levels in synovial fluid suggested local net conversion of cortisone to cortisol.

### Measures of systemic glucocorticoid metabolism in inflammatory arthritis

**Figure 5:**
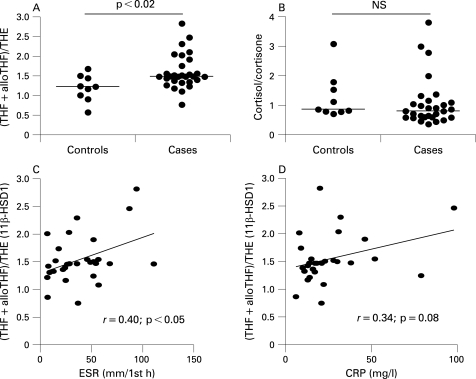
Systemic measures of local glucocorticoid metabolism in patients with rheumatoid arthritis (RA) and non-inflammatory joint disease. (A, B) Measurement of the balance of glucocorticoid activation/inactivation in non-inflammatory controls and patients with untreated RA. There was a significant increase in the tetrahydrocortisol + allo-tetrahydrocortisol/tetrahydrocortisone (THF+alloTHF)/THE ratio in patients with inflammatory arthritis, whereas the urinary free cortisol/cortisone ratio was unchanged. This indicated that 11β-hydroxysteroid dehydrogenase type 1 (11β-HSD1)-mediated glucocorticoid activation was enhanced in patients with RA. (C, D) In patients with RA the relationship between the (THF+alloTHF)/THE ratio, a systemic measure of 11β-HSD1 activity, and inflammatory markers was examined. The erythrocyte sedimentation rate (ESR), but not C-reactive protein (CRP), demonstrated a significant correlation with this measure, suggesting increasing 11β-HSD1-mediated glucocorticoid activation with increasing degrees of inflammation.

Systemic measures of 11β-HSD1 activity were assessed in subjects with untreated RA compared with patients with non-inflammatory joint disease. There was a significantly higher ratio of cortisol to cortisone metabolites in the urine of patients with RA, suggesting higher 11β-HSD1 activity (figs 5A and B). No change was seen in the ratio of urinary cortisol to cortisone indicating that renal 11β-HSD2 was unchanged. There was a positive correlation between 11β-HSD1 activity and ESR (but not CRP) in patients with RA (figs 5C and D). The urinary cortisol/cortisone ratio also correlated with ESR in patients with RA (*r* = 0.42, p<0.05) probably reflecting the contribution 11β-HSD1 makes to this ratio. There was no correlation between the urinary cortisol/cortisone ratio and CRP (*r* = 0.11, NS). There was no correlation between total urinary corticosteroid metabolites and ESR or CRP (*r* = 0.29 and 0.08, both NS).

## DISCUSSION

By a variety of measures, substantial glucocorticoid metabolism was identified in the joint. The net consequence was local glucocorticoid excess. In patients with RA, glucocorticoid generation within synovium, and systemic levels of active steroid, correlated with disease activity and had functional effects on the inflammatory response. Although this may form part of an endogenous mechanism for immunoregulation, a subset of synovial cells were able to inactivate glucocorticoids through expression of 11β-HSD2 and thus may be glucocorticoid resistant.

Soon after the initial use of cortisone and hydrocortisone for RA it was realised that these steroids were interconverted in vivo and that 11β-HSD activity was essential for conversion of cortisone to cortisol.^25^ This suggested that synovial tissue itself might metabolise corticosteroids. Initially, it was thought that patients with RA would have increased ability to inactivate hydrocortisone.^26^ Although the understanding of tissue steroid metabolism was rudimentary, bidirectional 11β-HSD activity was noted in rheumatoid synovial tissue^27^ and significant amounts of cortisol were generated from cortisone injected into inflamed joints.^28^ Recently, interest has been renewed, with reports implicating 11β-HSD2 expression in RA. 11β-HSD2 was the most upregulated of >4300 genes in a study examining PBMCs from patients with recent onset RA compared with longstanding RA.^18^ 11β-HSD2 was one of three (out of 20 000) significantly upregulated genes in PBMCs from identical twins discordant for RA.^17^ Analysis of rheumatoid synovium indicated that 11β-HSD2 was expressed predominantly in synovial macrophages. A separate study examined the capacity of synovial explants to inactivate steroids and described how this activity was higher in patients with RA.^16^ That study did not involve direct measurement of glucocorticoid activation and relied on 11β-HSD inhibitors with limited specificity. Furthermore, the association between local and systemic glucocorticoid metabolism was not assessed. To address this, we used specific enzyme assays and selective inhibitors to define the nature of glucocorticoid metabolising activity. We additionally examined possible consequences of corticosteroid metabolism on local and systemic levels of glucocorticoids.

The generation of substantial amounts of active glucocorticoids within the joint could account for the reason why therapeutic glucocorticoids are so effective at dampening flares of synovial inflammation. In the inflamed joint, cells will be exposed to both circulating glucocorticoid and glucocorticoids generated from circulating inactive precursors by cells expressing 11β-HSD1. Locally produced glucocorticoids will be free to diffuse into surrounding tissues. This could affect the integrity of adjacent connective tissue. The periarticular osteoporosis seen in RA is of multifactorial origin but high local glucocorticoid levels would suppress bone-forming ability and thus contribute to uncoupling of bone resorption from formation.^29^ High glucocorticoid levels would also be expected to have an immunosuppressive effect. This could contribute to the increased risk of septic arthritis in RA.^30^

The finding of 11β-HSD2 expression in synovium raises the possibility that some cells within synovium are resistant to steroids through expression of this enzyme. Further assessment of the functional implications of 11β-HSD2 expression is beyond the scope of this study but there is a need to define further the phenotype of 11β-HSD2-expressing synovial cells and to clarify the relationship these cells have with 11β-HSD2-positive PBMCs. An important and unexpected finding in this study is the presence of considerable glucocorticoid metabolising capacity in synovium from patients with OA. This tissue was initially used as a control for patients with RA with the expectation that glucocorticoid metabolism would be less prominent. However, net activity appeared similar to that of RA. Additionally, the relative decrease in IL6 production in response to cortisone treatment was similar. The inability of the inhibitor to block the capacity of OA synovium to convert cortisol to cortisone also suggests that 11β-HSD2 is expressed. 11β-HSD2 expression in OA has been identified in a previous study,^16^ which suggested that its expression is lower in OA than RA. The roles of 11β-HSD1/2 in OA remain to be defined.

Glucocorticoid activation was associated with ESR but not CRP measurements. A potential explanation for this is the confounding effect glucocorticoid activation had on synovial IL6 production. CRP synthesis is primarily an IL6-driven process and thus the relationship between joint inflammation and CRP will be complicated by the inhibitory effect of glucocorticoids on IL6. The ESR is less dependent on IL6 and so less likely to be directly influenced by local glucocorticoid metabolism.

The data presented here have several limitations. First, synovial tissue was obtained from patients with RA treated with various disease-modifying drugs. The small number of patients studied makes it difficult to comment about potential effects of antirheumatic treatment on steroid metabolising enzyme activity. Second, synovial tissue was examined in patients who had established RA. It will be of interest to study temporal changes in glucocorticoid metabolism in patients with RA at different disease stages and to assess whether findings are specific to RA or a general feature of persistent synovial inflammation. Although urinary measures of systemic steroid metabolism correlated well with measures in synovial tissue and fluid, an intrinsic limitation is that these might reflect altered steroid metabolism in tissues other than synovium. We have previously reported that inflammatory cytokines increase 11β-HSD1 expression in fibroblasts from several tissues^12^ and 11β-HSD1 expression has been reported in subpopulations of peritoneal macrophages^31^ and T cells.^32^ Regardless of the extent of altered glucocorticoid metabolism in RA, the functional consequences of this deserve further study.
